# The Role of BDNF in the Neuroimmune Axis Regulation of Mood Disorders

**DOI:** 10.3389/fneur.2019.00515

**Published:** 2019-06-04

**Authors:** Yang Jin, Li Hua Sun, Wei Yang, Ran Ji Cui, Song Bai Xu

**Affiliations:** ^1^Jilin Provincial Key Laboratory on Molecular and Chemical Genetic, Second Hospital of Jilin University, Changchun, China; ^2^Department of Neurosurgery, First Hospital of Jilin University, Changchun, China

**Keywords:** BDNF, neuroimmune axis, mood disorders, depression, inflammation, cytokines

## Abstract

The neuroimmune system plays a crucial role in the regulation of mood disorders. Moreover, recent studies show that brain-derived neurotrophic factor (BDNF), a member of the neurotrophin family, is a key regulator in the neuroimmune axis. However, the potential mechanism of BDNF action in the neuroimmune axis' regulation of mood disorders remains unclear. Therefore, in this review, we focus on the recent progress of BDNF in influencing mood disorders, by participating in alterations of the neuroimmune axis. This may provide evidence for future studies in this field.

## Introduction

Mood disorders are one of the most common mental disorders in the world, especially in western society. Epidemiological studies have found that there are approximately 350 million people affected by depression in the world, and the number is increasing year on year (1, 2). According to the results of the global burden of disease study, years lost to disability of depression ranks first among the top 10 disabling diseases in the world (3). The main clinical features are marked by consistent emotional upsurge or depression, often accompanied by corresponding changes in thinking and behavior (4). The performance of mood disorders is highly variable. Lighter ones may respond to certain negative life events, while heavier ones may become a seriously recurrent or even chronic disabling disorder. Clinically, mood disorders can be divided into four types: depressive episode, manic episode, bipolar disorder, and a persistent mood disorder. It not only brings severe mental pain to patients but also leads to other diseases, such as heart disease and cerebrovascular diseases. However, the pathogenesis of mood disorders is still unclear, so it is difficult for patients to be cured. Although mood disorders can currently be treated with drugs, psychotherapy or a combination thereof, the efficacy is limited and side effects may also occur (5, 6). It is therefore necessary to explore the etiology and mechanism of mood disorders to treat and prevent mood disorders.

There are many theories about the nosogenesis of depression disorder, such as monoamine neurotransmitter hypothesis, hypothalamus-pituitary-adrenal (HPA) axis dysfunction, neurotrophic hypothesis and cytokine hypothesis (7, 8). In recent years, more and more studies have focused on the relationship between mood disorders and neuroimmune regulation (9). Many neurotrophins are associated with the pathogenesis of mood disorders, such as the nerve growth factor (NGF) and brain-derived neurotrophic factor (BDNF) (10, 11). Several studies have shown that BDNF may be indispensable to the neuroimmune regulation of mood disorders. However, the potential mechanism for BDNF to affect mood disorders, by participating in changes in the neural-immune axis, has not been elucidated. In this review, we summarize the latest progress in the role of BDNF in the neuroimmune axis regulation of mood disorders. It may provide new ideas for the research and treatment of mood disorders in the future.

## BDNF and Mood Disorders

BDNF, an important member of the neurotrophic factor family, is a protein synthesized in the brain and widely distributed in the central nervous system (CNS), as well as the peripheral nervous system (12). It can promote the survival, growth, differentiation, and development of neurons and plays a crucial role in the neural structure and functional plasticity (13, 14). A large number of human and animal studies have implicated the close links between BDNF and the occurrence and treatment of various diseases, including schizophrenia (15), Alzheimer's disease (16, 17), mood disorders (18), and Parkinson's disease (19).

### Studies About BDNF in Depression

The neurotrophic hypothesis suggests that pathological changes in brain areas associated with depression, are closely related to BDNF expression and functional down-regulation (20). Animal models of depression suggest the vital function of BDNF in the pathophysiological mechanism of depression. In animal experiments, chronic stress and depression conditions decreased BDNF expression, increased apoptosis and decreased regeneration of neurons in the hippocampus, and also decreased BDNF expression in other parts of the brain (21, 22). However, whether these speculations apply to humans still remain to be tested in clinical studies. A large number of clinical studies have found that various kinds of stress can reduce the activity of the BDNF pathway in the hippocampus and prefrontal cortex (23–25). The postmortem studies of Karege et al. (26) reported that the analysis of brain tissue samples from patients with depression after self-killing found that BDNF and TrkB expression in the hippocampus decreased. Moreover, in the hippocampus of patients who have received antidepressant treatment before their death, BDNF and TrkB expression increased (26). The vast majority of studies have found abnormally lower serum BDNF levels in patients with depression than that of people without depression (25, 27, 28). Ristevsk-Dimitrovska et al. found that the serum BDNF level of depressed patients was lower than that of the control group (29), while significantly higher BDNF levels were found after antidepressant treatment (24). A meta-analysis showed that the serum or plasma BDNF increased during treatment in severe mental illness inpatients, but was not restored (18). Treatment with an antidepressant, Agomelatine, could increase the hippocampal BDNF level and BDNF positive neurons in CUMS rats (30). Kreinin et al. found that the serum BDNF level was positively correlated with depression in women with severe MDD, which further supported the role of BDNF in the pathogenesis and treatment of MDD (31). Moreover, increased BDNF levels suggests that BDNF may serve as a marker for a therapeutic response to ECT in MDD patients (32). Thus, it has been speculated that BDNF may be a biomarker of depression. But to understand the role of BDNF in depression, it is also necessary to further clarify the regulatory factors affecting BDNF expression, namely the upstream and downstream signaling pathways of BDNF in the nervous system.

### BDNF and Neurotransmitters in Depression

The monoaminergic hypothesis is also one of the most important hypotheses to study in the pathogenesis of depression. It points out that depression may be caused by low levels of monoamine neurotransmitters in the brain (33). Meanwhile, the function of BDNF is closely related to the plasticity of 5-HT, choline lipids, DA neurons and the survival of central neurons. For example, BDNF could promote the regeneration of 5-HT neurons in the CNS, so the large consumption of 5-HT in the CNS reduces the BDNF level, which leads to the atrophy and death of nerve cells, affects neural plasticity, and thus aggravates depressive symptoms (34). In addition, the regulatory effect of BDNF on the 5-HT_2A_ receptor level is also an important mechanism of BDNF's role in affective disorder (35). A study suggested that BDNF was implicated in the neuroprotective effects of the selective 5-HT_1A_ receptor agonist, 8-OH-DPAT, against CA1 neurons apoptotic death after transient global cerebral ischemia (36). BDNF also plays an essential role in the mesolimbic DA pathway. Studies have shown that the blockade of BDNF activity in the ventral tegmental area-nucleus accumbens pathway exerts an antidepressant-like activity in rodent models of stress (37–39). BDNF controls the expression of the D3 receptor in part of the brain, and induction of BDNF by antidepressant treatments is associated with its behavioral activity (40).

### Signaling Pathway of BDNF in Depression

**Figure 1:**
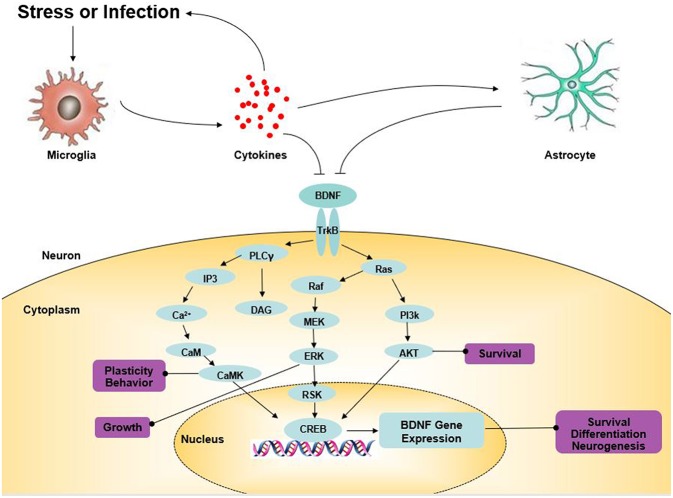
The role of BDNF in depression. Arrows indicate activation; T-shaped arrows indicate inhibition. Akt, serine/threonine protein kinase; BDNF, brain-derived neurotrophic factor; CaM, calmodulin; CaMK, calcium-calmodulin-dependent protein kinase; CREB, cAMP response element-binding protein; DAG, diacylglycerol; ERK, extracellular signal-regulated kinase; IP3, inositol 1,4,5-trisphosphate; MEK, mitogen-activated extracellular signal-regulated kinase; PKC, protein kinase C; PI3K, PI-3 kinase; PLC-γ, phospholipase-Cγ; RSK, ribosomal S6 kinase; TrkB, tyrosine kinase B.

Tyrosine receptor kinase B (TrkB), a member of the tyrosine kinase family, can specifically bind to BDNF with a high affinity (41). Comprehensive research has indicated that BDNF is involved in the regulation of CNS, mainly by binding to TrkB (Figure 1). BDNF activates intracellular tyrosine kinase activity by binding to TrkB, causing the autophosphorylation of TrkB, thereby activating the mitogen-activated protein kinase (MAPK) pathway, the phospholipase C-gamma (PLC-γ) pathway, the phosphatidylinositol 3-kinase (PI3K) pathway, and other signaling pathways (42). Finally, CREB is activated at the Ser133 site of the cAMP response element binding protein (CREB). CREB promotes the survival of nerve cells and increases synaptic plasticity and neurogenesis by boosting the expression of the BDNF and BCL-2 genes (43). BDNF-TrkB not only affects the survival, development, and functions of neurons but also promotes the formation of the dendritic spine, provides a structural basis for synapse formation and improves the transmission efficiency of synapses. BDNF/TrkB signaling has a major impact on the production of antidepressant effects (44). Knock-out of the BDNF gene or the reduction of the levels of BDNF in the forebrain blocks the behavioral effects of antidepressants (45). In recent years, more and more studies have found that antidepressants might play an anti-depressant role by up-regulating brain BDNF levels or activating TrkB receptors. Song et al. found that silibinin mitigated the depression-like symptoms of Aβ1-42-treated rats by decreasing the BDNF/TrkB expression, suggesting the role of the BDNF/TrkB signaling pathway in the activity of antidepressants (46). Likewise, sesquiterpenoids from ginseng root treatment, ameliorate depression-like behaviors induced by LPS by upregulating the BDNF/TrkB Pathway (47). The BDNF-TrkB pathway in the nucleus accumbens of α7 nACHR knockout mice was demonstrated to be up-regulated, which was considered to be involved in their depression-like behavior (48). The antidepressant role of fisetin was confirmed by Wang et al. which was achieved by activating TrkB rather than regulating its overall level (49). All of these studies suggest that BDNF and the mediated TrkB signaling pathway may provide new approaches for the treatment of depression.

The N-methyl-D-aspartic acid receptor (NMDA receptor) is associated with depression. NMDA receptor antagonism has a significant antidepressant effect. On the one hand, it exerts its antidepressant effect by inhibiting NMDA receptors, which not only promote the establishment of new synaptic connections but also restore the synaptic connections caused by stress damage. On the other hand, antagonizing the NMDA receptor also activates the AMPA receptor, which provides a fast antidepressant effect through its signaling pathway. This provides a new method in the treatment of traditional depression (50). BDNF may play a role through the NMDA receptor. BDNF enhances the AMPA-dependent synaptic signaling in the hippocampus through downstream pathways mediated by NMDA receptors (51). Wang et al. enhanced the BDNF/TrkB signaling pathway by means of transcranial magnetic stimulation (TMS). At the same time, the activity of the NMDA receptor in the cerebral cortex was strongly correlated with the degree of TrkB activation (52). In cultured hippocampal neurons and rat neocortical cells, the activation of TrkB or chronic administration of BDNF can enhance the expression of the NMDA receptor NR1 and NR2A/2B through transcriptional activation. BDNF can also promote the release of glutamate through the presynaptic receptor signal transduction pathway, and enhance the AMPA receptor and NMDA through the postsynaptic receptor pathway and then participate in and promote the formation of LTP (53). Duncan et al. through a study of 30 depressive patients treated with ketamine, found that the antidepressant effect of ketamine might be due to the enhancement of inter-synaptic communication by BDNF (54).

### Effects of Antidepressants on BDNF

In recent years, the major clinical antidepressants are monoamine oxidase inhibitors, tricyclic inhibitors, and tetracyclic inhibitors (55, 56). Some clinical studies suggest that antidepressant therapy for a period of time could reverse the decrease of peripheral BDNF levels of depressed patients. Serum BDNF levels of depressed patients taking SSRIs were markedly higher than that of the control group and depressed patients not taking SSRIs (57). Treatment with venlafaxine or paroxetine also increased BDNF in patients with depression (58). However, whether all antidepressants affect BDNF levels, remains controversial. A meta-analysis showed that the level of peripheral BDNF increased during antidepressant treatment of SSRIs and SNRIs, among which sertraline could improve the BDNF level after short-term treatment (59). Treatment with fluoxetine (SSRI) was found to alter BDNF levels in patients with depression, whereas venlafaxine did not (60). Freire et al. found that neither the combined group, nor the pharmacological group resulted in the increase of the serum BDNF level in patients with depression, although both significantly improved the depressive symptoms of patients (61). These studies indicate that different antidepressants may have different effects on the peripheral BDNF during treatment.

## Neuro-Immune Axis and Mood Disorders

### Neuro-Immune Axis

Nowadays, plenty of research has been conducted on the interaction between the central nervous system and immune system. It is neurogenic inflammation that determines whether the immune response is caused by a local threat, through the connection of nerve fibers to immune cells. In recent research, a CNS with complicated innate immune responses is demonstrated to have high immunocompetence (62). Microglia, resident immune cells within the brain parenchyma, can secrete some soluble factors, such as chemokines, cytokines, and neurotrophic factor, to adjust the CNS immune response and tissue repair (63). In addition, astrocytes also play an essential role in central immunity. They respond to an inflammatory environment not only in an immunological way by changing their cell phenotype, but also modulate the immune response of lymphocyte in the brain by releasing associated protein molecules, like chemokines and cytokines (64).

Some immune cells, such as non-specific leucocytes and lymphocytes, produce neurotransmitters and neuropeptides. Opioids may serve as an example. It is suggested that opioids are secreted in inflammatory tissues and act to alleviate clinical pain under stress by activating peripheral opioid receptors (65). There are also some neurotrophins produced by activated lymphocytes, such as BNDF and NGF (66, 67). In turn, non-specific leucocytes and lymphocytes can also express classic neuronal receptors. For example, the activated non-neuron A7 nicotinic cholinergic receptor has anti-inflammatory and immunomodulatory effects on multiple cell types, T cells, B cells, dendritic cells, and mononuclear phagocytes included (68).

Cytokines, chemokines and their receptors were reported to express on the central and peripheral nervous systems. For instance, IFN-α not only affected CNS directly but also had an indirect action through inflammatory cytokines of the central and peripheral nervous systems (69). Previous studies found that interleukin and chemokine receptors, which participated in neuronal inflammation and CNS diseases, were expressed by neurons (70). Cytokines like interleukin-1β (IL-1β), interleukin-6 (IL-6) and tumor necrosis factor-α (TNF-α) could influence the behavior, by directly functioning in the nervous system. Cytokines are conducive to the growth and function of the brain and regulate neural activity and neurotransmitter systems, which result in behavioral changes. Chronic exposure to high levels of inflammatory cytokines and constant alterations of central neurotransmitters may contribute to psychiatric disorders like schizophrenia and mood disorders (71, 72). Cytokines induce behavioral effects by activating inflammatory signaling pathways in the brain, leading to the reduction of growth factors such as BDNF for instance (72).

### Cytokines and Depression

In recent years, studies have found that immune dysfunction was closely related to depression, and pro-inflammatory cytokines produced by innate immune activation were especially closely related to the occurrence and development of depression. Therefore, the hypothesis of cytokine is gradually proposed. The cytokine hypothesis suggests that depression is an inflammatory disease caused by neuroimmune regulation disorders, emphasizing that the body's immune system plays an important role in depression. Cytokines are intercellular information transfer molecules, which mainly have immunomodulatory and multiple effector functions. Different cytokines play different roles in inflammation. Some have proinflammatory effects, while others have anti-inflammatory effects. For instance, IL-1β, IL-6, and tumor necrosis factor α (TNF-α) are relatively advanced proinflammatory cytokines, while IL-4 and IL-10 is the main area of research in anti-inflammatory cytokines. Clinical studies have shown that patients with depression were often associated with varying degrees of inflammatory activation or increased inflammatory molecules, suggesting that the occurrence of depression might be closely related to cytokines (73, 74).

#### Studies About Cytokines in Depression

This hypothesis has been supported by a large number of clinical cases in recent years: patients with autoimmune diseases and chronic viral infections often showed depressive symptoms (75, 76). Autopsy studies have found that cytokines were significantly increased, as well as the synthesis of carbon monoxide synthase in macrophages, microglia, and astrocyte (77). A number of studies have indicated that various cytokines, such as IL-1β, IL-2, IL-6, TNF-α, and IFN-γ in serum or plasma of patients with depression were significantly increased (78). The results of the meta-analysis showed that the levels of inflammatory factors in patients with depression, including IL-1, IL-6, and TNF-α, were significantly higher than those of healthy people and in positive relevance to the serious extent of depressive symptoms (79–81), and antidepressants could lower these cytokines in people with depression (82). According to the study of the cytokine overview, Zou et al. found that the expression of IL-1, IL-10 and TNF in MDD patients increased significantly, while the expression of IL-8 decreased significantly. Such aberrant changes in the levels of inflammatory cytokines demonstrated that it is depression that activates the inflammatory process (83). Animal studies have also shown that the levels of IL-1β, IL-6, and TNF-α in the brain increased significantly after lipopolysaccharide treatment, as well as depressive behaviors, such as sleep disorders, loss of pleasure, and insufficiency of power (84). Depressive behaviors of animals were blocked after injecting IL-1 receptor antagonist IL-1rA into animals before stress (85). The results suggested that inflammatory pathways might be involved in the development of depression.

#### Actions of Cytokines in Depression

People realize that there is a two-way effect between immunity and nerves. Both physiological and psychological stress can activate the immune system and make the cytokines secreted, and then influence the central nervous system, such as neurogenesis, neurotransmitter level, neuroendocrine function, neuroplasticity, and behavior related neural pathways (86–88). It leads to changes in the neurochemistry and endocrinology associated with depression and influences the development of depression. Cytokines can promote oxidative stress and damage glial cells in emotionally related brain regions, such as the prefrontal cortex and amygdala (89). In addition, dysfunctions of glutamate-induced by cytokine can reduce the generation of neurotrophic factors (88). Under stress, the increase of proinflammatory cytokines in the human body activates the indolamine 2,3- dioxygenase (IDO), an enzyme that can directly act on the metabolism of tryptophan (TRP). IDO can increase the level of kynurenine produced by TRP metabolism, thereby reducing the level of 5-HT and promoting the occurrence of depression (90). Cytokines are also momentous to the dysfunction of the HPA axis. They induce the hyperactivity of the HPA axis to increase the glucocorticoid for a long time, and the abnormal glucocorticoid signal can affect the production, maintenance, and development of depressive behaviors (91). At the same time, these cytokines and excess glucocorticoids also inhibit nerve regeneration in the brain. Cytokine signaling pathways, for example NF-kB, can disrupt the function and expression of glucocorticoid receptors, leading to an unrestricted inflammatory response, further exacerbating depressive symptoms (92). Additionally, cytokines can also contribute to depression by influencing neural plasticity. Ben et al. confirmed that IL-1 could inhibit neuronal regeneration, and inflammatory cytokines—IL-6, as an example, could disrupt neuronal function (93). Currently, though the causal relationship between cytokines and depression is still considered controversial, it is undeniable that the negative regulation of neuroplasticity in the brain has a significant impact on the developmental progress of depression. Furthermore, the study also shows that abnormally changed levels of cytokines are associated with an increased risk of delirium and suicide (94–96).

### Immune Regulation and Antidepressant Effects

Some anti-inflammatory drugs have antidepressant effects or enhance antidepressant effects. Studies have shown that the anti-inflammatory drug COX-2 inhibitors could directly or indirectly affect the 5-HT system through the CNS and play an antidepressant effect. Giving rats a dose of rofecoxib can increase the level of 5-HT in the prefrontal and parietal cortex (97). Celecoxib in depressed rats was found to decrease cytokine levels and improve behavior in the hypothalamus (98). Through the drug combinations of celecoxib with antidepressants, such as reboxetine, fluoxetine, and sertraline, it was found that the combined group was better than an antidepressant alone in patients with depression (99–101). Etanercept, a kind of tumor necrosis factor (TNF- α) antagonist, also has a strong antidepressant effect, which can improve depression symptoms and patient fatigue (102). Many other clinical cases show that antidepressants can reduce proinflammatory cytokines and other inflammatory markers in patients (103). Tricyclic antidepressants, SSRI, SNRI, and other antidepressants have been shown to increase anti-inflammatory immunomodulatory cytokine levels by inhibiting inflammatory cytokines and th1-like cytokines (such as IFN-γ). Réus et al. revealed that imipramine, an antidepressant, could reduce the levels of TNF-α and IL-1β in cerebrospinal fluid of maternally deprived adult rats (104). Studies have shown that some non-pharmacological treatments can also improve depressive symptoms by regulating immune inflammatory pathways. Kim et al. believe that acupuncture can reduce the levels of peripheral and central proinflammatory factors (IL-1, IL-6, TNF- α) and proinflammatory neuropeptides, and the results are better in the treatment of depression (105). In addition, exercise can play a synergistic role by inhibiting the immune and inflammatory pathways (106).

## BDNF, Neuroimmune Axis, and Mood Disorders

BDNF is a relatively mature neurotrophic factor, which can promote the proliferation of neurons and glial cells in the inflammation of the nervous system through various molecular mechanisms (107, 108). Glial cells are innate immune cells in the center. They not only synthesize and release multiple inflammatory mediators but also express many inflammatory mediator receptors on the cell surface. Microglia, the first line of defense for the central immune response, exerts essential influence on the inflammatory response in the brain (109). Although there is no direct evidence that microglia are correlated with the nosogenesis of depression, many studies have examined if there is a significant increase in the amount of microglia in the brain in patients with suicidal depression. A previous study observed microgliosis in the dorsolateral prefrontal cortex, anterior cingulate cortex, mediodorsal thalamus, and hippocampus of suicidal patients (110). Torres-Platas et al. also observed a relative increase of primed microglia in depressed suicides (77). Microglia can regulate the release of BDNF. Microglia may take effect on pathogenesis by reducing BDNF expression as well as its high-affinity receptor TrkB. Studies demonstrated that microglia was of extensive and diverse importance for the formation of appropriate synaptic connections during development and maturation, which were frequently mediated by BDNF (111). In addition, high levels of IL-6, IL-1β, and BDNF in LPS-stimulated normal human astrocytes (NHAs) was observed, using an LPS-induced *in vitro* injury model of astroglial cultures. Vice versa, BDNF can promote the growth of astrocytes and regulate the viability and proliferation of LPS-induced NHA through the PI3K/AKT pathway (112, 113).

Reports repeatedly demonstrated that inflammatory cytokines affect neuronal development as well as apoptosis (114, 115). As a matter of fact, stress and its associated activation of inflammatory cytokines might have a negative effect on neurogenesis and neuroplasticity (84, 116, 117). Considerable research efforts have been devoted to the effect of inflammation on the BDNF expression in the brain. The significant reduction in BDNF was caused by the administration of pro-inflammatory cytokines or lipopolysaccharide (LPS), an inducer for cytokines, serve as an example. LPS injections could significantly reduce mature BDNF levels in the hippocampus and cerebral cortex (118), as well as IFN-α administration, which decreased systemic BDNF levels (119). Furthermore, other neurotrophic factors also decreased to varying degrees: NGF and neurotrophic factor−3 (NT-3), for instance (120).

It was demonstrated in a number of research studies that inflammation inhibits BDNF/TrkB expression. Inflammatory cytokines influence the phosphorylation of the BDNF receptor (TrkB), thereby further interfering with BDNF signaling (121). Gibney et al. found that poly-I:C administration upregulated the expression of the inflammatory cytokines, which caused the occurrence of an inflammatory reaction. At the same time, BDNF and TrkB expression in the hippocampus and cortex were downregulated, which might lead to behavioral defects of depression and anxiety (122). In addition, it is under integrated BDNF signaling that antidepressants are able to reverse LPS-induced apoptosis, which agrees well with the above-mentioned studies.

The anti-inflammatory mechanism of antidepressive agents has not been elucidated yet. Imipramine has been shown to suppress proinflammatory cytokines in rat neural stem cells, stimulating the expression of BDNF (123). Studies have shown that the production of inflammatory cytokines was regulated by complex signaling pathways, especially the nuclear factor-κb (NF-κB) inflammatory response signal pathway (BDNF-TrkB-MEK-ERK-NF-κB pathway) whose activation plays a central regulatory role in the inflammatory response. Investigation of the effect and potential mechanism of salidroside on depression showed that salidroside could down-regulate the expression of BDNF, TrkB, and the NF-κB protein (124). Ge et al. thought that the antidepressant effect of resveratrol is mainly to reduce the expression of inflammatory cytokines and improve NF-κB activation (125). Chrysophanol could inhibit the NF-κB signaling pathway (126), and the high dose of fisetin could regulate the expression of NF-κB in the hippocampus to antagonize the expression of iNOS mRNA (127). Similarly, the antidepressant effect of aesculetin may be achieved by inhibiting the NF-κB pathway as well as activating BDNF/TrkB signaling (128). Furthermore, as an inflammatory intracellular signaling molecule, p38 mitogen-activated protein kinase is now a target for clinical studies of chronic inflammatory diseases due to the potential antidepressant effects of its inhibitors (129). All these studies provide a basis for the development of new clinical antidepressants and the continued development of antidepressant treatments.

## Conclusion

**Figure 2:**
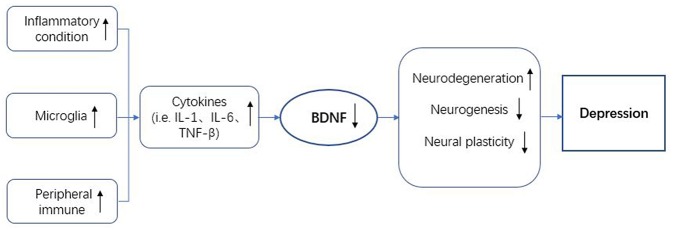
BDNF in the neuroimmune regulation axis of depression. BDNF, brain-derived neurotrophic factor; IL-1, interleukin-1; IL-6, interleukin-6; TNF-β, tumor necrosis factor-β.

Based on many clinical and basic research studies, a variety of theories were proposed to expound the nosogenesis of mood disorders, especially depression. In this review, the neuroimmune axis has been related to mood disorders (Figure 2). BDNF is thought to be involved in the neuroimmune axis regulation. On the one hand, the expression of BDNF is affected by immune cells and the immune factors they secrete. On the other hand, the immunomodulatory process also requires the regulation of BDNF-mediated signaling pathways. Unfortunately, the specific mechanism of how BDNF participates in the regulation of the neuroimmune axis in mood disorders is still unclear and it is therefore necessary to conduct more in-depth research. The treatment of mood disorders in the past often only focus on a certain aspect of research. The characteristics of the varied symptoms of depression determine that these treatments are not effective. Exploring a treatment strategy for depression based on neuroimmune axis regulation may be more helpful to further guide the development of anti-mood disorders drugs.

## Author Contributions

YJ and RJC contributed conception and design of the review. WY organized the documents. YJ wrote the first draft of the manuscript. SBX and LHS wrote sections of the manuscript. All authors contributed to manuscript revision, read and approved the submitted version.

### Conflict of Interest Statement

The authors declare that the research was conducted in the absence of any commercial or financial relationships that could be construed as a potential conflict of interest.

## References

[1] Zhao X Sun L Sun YH Ren C Chen J Wu ZQ. Association of HTR2A T102C and A-1438G polymorphisms with susceptibility to major depressive disorder: a meta-analysis. Neurol Sci. (2014) 35:1857–66. 10.1007/s10072-014-1970-7 25270656

[2] Duman RS Aghajanian GK Sanacora G Krystal JH. Synaptic plasticity and depression: new insights from stress and rapid-acting antidepressants. Nat Med. (2016) 22:238–49. 10.1038/nm.4050 26937618 PMC5405628

[3] Smith K. Mental health: a world of depression. Nature. (2014) 515:181. 10.1038/515180a 25391942

[4] Cui R. Editorial: a systematic review of depression. Curr Neuropharmacol. (2015) 13:480. 10.2174/1570159X1304150831123535 26412067 PMC4790400

[5] Segal ZV Dinh-Williams LA. Mindfulness-based cognitive therapy for relapse prophylaxis in mood disorders. World Psychiatry. (2016) 15:289–91. 10.1002/wps.20352 27717259 PMC5032518

[6] Rodrigues MF Nardi AE Levitan M. Mindfulness in mood and anxiety disorders: a review of the literature. Trends Psychiatry Psychother. (2017) 39:207–15. 10.1590/2237-6089-2016-0051 28767927

[7] Ogłodek E Szota A Just M Moś D Araszkiewicz A. The role of the neuroendocrine and immune systems in the pathogenesis of depression. Pharmacol Rep. (2014) 66:776–81. 10.1016/j.pharep.2014.04.009 25149980

[8] Gardner A Boles RG. Beyond the serotonin hypothesis: mitochondria, inflammation and neurodegeneration in major depression and affective spectrum disorders. Prog Neuropsychopharmacol Biol Psychiatry. (2011) 35:730–43. 10.1016/j.pnpbp.2010.07.030 20691744

[9] Hodes GE Kana V Menard C Merad M Russo SJ. Neuroimmune mechanisms of depression. Nat Neurosci. (2015) 18:1386–93. 10.1038/nn.4113 26404713 PMC4843114

[10] Castrén E Kojima M. Brain-derived neurotrophic factor in mood disorders and antidepressant treatments. Neurobiol Dis. (2017) 97:119–26. 10.1016/j.nbd.2016.07.010 27425886

[11] Wiener CD de Mello Ferreira S Pedrotti Moreira F Bittencourt G de Oliveira JF Lopez Molina M. Serum levels of nerve growth factor (NGF) in patients with major depression disorder and suicide risk. J Affect Disord. (2015) 184:245–8. 10.1016/j.jad.2015.05.067 26118751

[12] Martinowich K Lu B. Interaction between BDNF and serotonin: role in mood disorders. Neuropsychopharmacology. (2008) 33:73–83. 10.1038/sj.npp.1301571 17882234

[13] Nemcsik J László A Lénárt L Eörsi D Torzsa P Korösi B. Hyperthymic affective temperament and hypertension are independent determinants of serum brain-derived neurotrophic factor level. Ann Gen Psychiatry. (2016) 15:17. 10.1186/s12991-016-0104-4 27478486 PMC4966794

[14] Kowianski P Lietzau G Czuba E Waśkow M Steliga A Moryś J. BDNF: a key factor with multipotent impact on brain signaling and synaptic plasticity. Cell Mol Neurobiol. (2018) 38:579–93. 10.1007/s10571-017-0510-4 28623429 PMC5835061

[15] Islam F Mulsant BH Voineskos AN Rajji TK. Brain-derived neurotrophic factor expression in individuals with schizophrenia and healthy aging: testing the accelerated aging hypothesis of schizophrenia. Curr Psychiatry Rep. (2017) 19:36. 10.1007/s11920-017-0794-6 28534294

[16] Nagata T Kobayashi N Shinagawa S Yamada H Kondo K Nakayama K Plasma BDNF levels are correlated with aggressiveness in patients with amnestic mild cognitive impairment or Alzheimer disease. J Neural Transm. (2014) 121:433–41. 10.1007/s00702-013-1121-y 24253237

[17] Song JH Yu JT Tan L. Brain-derived neurotrophic factor in Alzheimer's disease: risk, mechanisms, and therapy. Mol Neurobiol. (2015) 52:1477–93. 10.1007/s12035-014-8958-4 25354497

[18] Nuernberg GL Aguiar B Bristot G Fleck MP Rocha NS. Brain-derived neurotrophic factor increase during treatment in severe mental illness inpatients. Transl Psychiatry. (2016) 6:e985. 10.1038/tp.2016.227 27959329 PMC5290335

[19] Fischer DL Auinger P Goudreau JL Paumier KL Cole-Strauss A Kemp CJ. Bdnf variant is associated with milder motor symptom severity in early-stage Parkinson's disease. Parkinsonism Relat Disord. (2018) 53:70–5. 10.1016/j.parkreldis.2018.05.003 29759928

[20] Kozisek ME Middlemas D Bylund DB. Brain-derived neurotrophic factor and its receptor tropomyosin-related kinase B in the mechanism of action of antidepressant therapies. Pharmacol Ther. (2008) 117:30–51. 10.1016/j.pharmthera.2007.07.001 17949819

[21] Filho CB Jesse CR Donato F Giacomeli R Del Fabbro L da Silva Antunes M. Chronic unpredictable mild stress decreases BDNF and NGF levels and Na (+),K (+)-ATPase activity in the hippocampus and prefrontal cortex of mice: antidepressant effect of chrysin. Neuroscience. (2015) 289:367–80. 10.1016/j.neuroscience.2014.12.048 25592430

[22] Kubera M Obuchowicz E Goehler L Brzeszcz J Maes M. In animal models, psychosocial stress-induced (neuro)inflammation, apoptosis and reduced neurogenesis are associated to the onset of depression. Prog Neuropsychopharmacol Biol Psychiatry. (2011) 35:744–59. 10.1016/j.pnpbp.2010.08.026 20828592

[23] Yoshida T Ishikawa M Niitsu T Nakazato M Watanabe H Shiraishi T Decreased serum levels of mature brain-derived neurotrophic factor (BDNF), but not its precursor proBDNF, in patients with major depressive disorder. PLoS ONE. (2012) 7:e42676 10.1371/journal.pone.0042676 22880079 PMC3411809

[24] Sen S Duman R Sanacora G. Serum brain-derived neurotrophic factor, depression, and antidepressant medications: meta-analyses and implications. Biol Psychiatry. (2008) 64:527–32. 10.1016/j.biopsych.2008.05.005 18571629 PMC2597158

[25] Taliaz D Stall N Dar DE Zangen A. Knockdown of brain-derived neurotrophic factor in specific brain sites precipitates behaviors associated with depression and reduces neurogenesis. Mol Psychiatry. (2010) 15:80–92. 10.1038/mp.2009.67 19621014 PMC2834321

[26] Karege F Vaudan G Schwald M Perroud N La Harpe R. Neurotrophin levels in postmortem brains of suicide victims and the effects of antemortem diagnosis and psychotropic drugs. Brain Res Mol Brain Res. (2005) 136:29–37. 10.1016/j.molbrainres.2004.12.020 15893584

[27] Cunha AB Frey BN Andreazza AC Goi JD Rosa AR Gonçalves CA. Serum brain-derived neurotrophic factor is decreased in bipolar disorder during depressive and manic episodes. Neurosci Lett. (2006) 398:215–9. 10.1016/j.neulet.2005.12.085 16480819

[28] Molendijk ML Spinhoven P Polak M Bus BA Penninx BW Elzinga BM. Serum BDNF concentrations as peripheral manifestations of depression: evidence from a systematic review and meta-analyses on 179 associations (N = 9484). Mol Psychiatry. (2014) 19:791–800. 10.1038/mp.2013.105 23958957

[29] Ristevska-Dimitrovska G Shishkov R Gerazova VP Vujovik V Stefanovski B Novotni A. Different serum BDNF levels in depression: results from BDNF studies in FYR Macedonia and Bulgaria. Psychiatr Danub. (2013) 25:123–7. 23793275

[30] Lu Y Ho CS McIntyre RS Wang W Ho RC. Agomelatine-induced modulation of brain-derived neurotrophic factor (BDNF) in the rat hippocampus. Life Sci. (2018) 210:177–84. 10.1016/j.lfs.2018.09.003 30193943

[31] Kreinin A Lisson S Nesher E Schneider J Bergman J Farhat K. Blood BDNF level is gender specific in severe depression. PLoS ONE. (2015) 10:e0127643. 10.1371/journal.pone.0127643 26010085 PMC4444333

[32] Rocha RB Dondossola ER Grande AJ Colonetti T Ceretta LB Passos IC. Increased BDNF levels after electroconvulsive therapy in patients with major depressive disorder: a meta-analysis study. J Psychiatr Res. (2016) 83:47–53. 10.1016/j.jpsychires.2016.08.004 27552533

[33] Shabbir F Patel A Mattison C Bose S Krishnamohan R Sweeney E. Effect of diet on serotonergic neurotransmission in depression. Neurochem Int. (2013) 62:324–9. 10.1016/j.neuint.2012.12.014 23306210

[34] Racagni G Popoli M. Cellular and molecular mechanisms in the long-term action of antidepressants. Dialogues Clin Neurosci. (2008) 10:385–400. 19170396 10.31887/DCNS.2008.10.4/gracagni PMC3181899

[35] Trajkovska V Santini MA Marcussen AB Thomsen MS Hansen HH Mikkelsen JD. BDNF downregulates 5-HT (2A) receptor protein levels in hippocampal cultures. Neurochem Int. (2009) 55:697–702. 10.1016/j.neuint.2009.06.013 19563850

[36] Salazar-Colocho P Del Río J Frechilla D. Neuroprotective effects of serotonin 5-HT 1A receptor activation against ischemic cell damage in gerbil hippocampus: Involvement of NMDA receptor NR1 subunit and BDNF. Brain Res. (2008) 1199:159–66. 10.1016/j.brainres.2007.12.032 18269931

[37] Eisch AJ Bolaños CA de Wit J Simonak RD Pudiak CM Barrot M. Brain-derived neurotrophic factor in the ventral midbrain-nucleus accumbens pathway: a role in depression. Biol Psychiatry. (2003) 54:994–1005. 10.1016/j.biopsych.2003.08.003 14625141

[38] Wook Koo J Labonté B Engmann O Calipari ES Juarez B Lorsch Z. Essential role of mesolimbic brain-derived neurotrophic factor in chronic social stress-induced depressive behaviors. Biol Psychiatry. (2016) 80:469–78. 10.1016/j.biopsych.2015.12.009 26858215 PMC4909591

[39] Berton O McClung CA Dileone RJ Krishnan V Renthal W Russo SJ. Essential role of BDNF in the mesolimbic dopamine pathway in social defeat stress. Science. (2006) 311:864–8. 10.1126/science.1120972 16469931

[40] Leggio GM Salomone S Bucolo C Platania C Micale V Caraci F. Dopamine D (3) receptor as a new pharmacological target for the treatment of depression. Eur J Pharmacol. (2013) 719:25–33. 10.1016/j.ejphar.2013.07.022 23872400

[41] Tejeda GS Díaz-Guerra M. Integral characterization of defective BDNF/TrkB signalling in neurological and psychiatric disorders leads the way to new therapies. Int J Mol Sci. (2017) 18:268. 10.3390/ijms18020268 28134845 PMC5343804

[42] Numakawa T Suzuki S Kumamaru E Adachi N Richards M Kunugi H. BDNF function and intracellular signaling in neurons. Histol Histopathol. (2010) 25:237–58. 10.14670/HH-25.237 20017110

[43] Takano K Yamasaki H Kawabe K Moriyama M Nakamura Y. Imipramine induces brain-derived neurotrophic factor mRNA expression in cultured astrocytes. J Pharmacol Sci. (2012) 120:176–86. 10.1254/jphs.12039FP 23076128

[44] Adachi M Autry AE Mahgoub M Suzuki K Monteggia LM. TrkB signaling in dorsal raphe nucleus is essential for antidepressant efficacy and normal aggression behavior. Neuropsychopharmacology. (2017) 42:886–94. 10.1038/npp.2016.201 27634357 PMC5312065

[45] Lepack AE Fuchikami M Dwyer JM Banasr M Duman RS. BDNF release is required for the behavioral actions of ketamine. Int J Neuropsychopharmacol. (2014) 18:pyu033. 10.1093/ijnp/pyu033 25539510 PMC4368871

[46] Song X Liu B Cui L Zhou B Liu W Xu F. Silibinin ameliorates anxiety/depression-like behaviors in amyloid β-treated rats by upregulating BDNF/TrkB pathway and attenuating autophagy in hippocampus. Physiol Behav. (2017) 179:487–93. 10.1016/j.physbeh.2017.07.023 28735062

[47] Wang W Liu X Liu J Cai E Zhao Y Li H. Sesquiterpenoids from the root of panax ginseng attenuates lipopolysaccharide-induced depressive-like behavior through the brain-derived neurotrophic factor/tropomyosin-related kinase B and sirtuin type 1/nuclear factor-κB signaling pathways. J Agric Food Chem. (2018) 66:265–71. 10.1021/acs.jafc.7b04835 29237268

[48] Zhang JC Yao W Ren Q Yang C Dong C Ma M. Depression-like phenotype by deletion of α7 nicotinic acetylcholine receptor: role of BDNF-TrkB in nucleus accumbens. Sci Rep. (2016) 6:36705. 10.1038/srep36705 27821848 PMC5099687

[49] Wang Y Wang B Lu J Shi H Gong S Wang Y. Fisetin provides antidepressant effects by activating the tropomyosin receptor kinase B signal pathway in mice. J Neurochem. (2017) 143:561–8. 10.1111/jnc.14226 28945929

[50] Zarate C Duman RS Liu G Sartori S Quiroz J Murck H. New paradigms for treatment-resistant depression. Ann N Y Acad Sci. (2013) 1292:21–31. 10.1111/nyas.12223 23876043 PMC3936783

[51] Burnouf S Martire A Derisbourg M Laurent C Belarbi K Leboucher A. NMDA receptor dysfunction contributes to impaired brain-derived neurotrophic factor-induced facilitation of hippocampal synaptic transmission in a Tau transgenic model. Aging Cell. (2013) 12:11–23. 10.1111/acel.12018 23082852

[52] Wang HY Crupi D Liu J Stucky A Cruciata G Di Rocco A. Repetitive transcranial magnetic stimulation enhances BDNF-TrkB signaling in both brain and lymphocyte. J Neurosci. (2011) 31:11044–54. 10.1523/JNEUROSCI.2125-11.2011 21795553 PMC3161730

[53] Carvalho AL Caldeira MV Santos SD Duarte CB. Role of the brain-derived neurotrophic factor at glutamatergic synapses. Br J Pharmacol. (2008) 153(Suppl. 1):S310–24. 10.1038/sj.bjp.0707509 18059328 PMC2268077

[54] Duncan WC Sarasso S Ferrarelli F Selter J Riedner BA Hejazi NS. Concomitant BDNF and sleep slow wave changes indicate ketamine-induced plasticity in major depressive disorder. Int J Neuropsychopharmacol. (2013) 16:301–11. 10.1017/S1461145712000545 22676966 PMC3510337

[55] López-Muñoz F Alamo C Juckel G Assion HJ. Half a century of antidepressant drugs: on the clinical introduction of monoamine oxidase inhibitors, tricyclics, and tetracyclics. Part I: monoamine oxidase inhibitors. J Clin Psychopharmacol. (2007) 27:555–9. 10.1097/jcp.0b013e3181bb617 18004120

[56] Fangmann P Assion HJ Juckel G González CA López-Muñoz F. Half a century of antidepressant drugs: on the clinical introduction of monoamine oxidase inhibitors, tricyclics, and tetracyclics. Part II: tricyclics and tetracyclics. J Clin Psychopharmacol. (2008) 28:1–4. 10.1097/jcp.0b013e3181627b60 18204333

[57] van der Meij A Comijs HC Dols A Janzing JG Oude Voshaar RC. BDNF in late-life depression: effect of SSRI usage and interaction with childhood abuse. Psychoneuroendocrinology. (2014) 43:81–9. 10.1016/j.psyneuen.2014.02.001 24703173

[58] Shen X Qian M Yuan Y Sun J Zhong H Yang J. [Research on association of BDNF gene Val66Met polymorphism with efficacy of antidepressants and plasma BDNF level]. Zhonghua Yi Xue Yi Chuan Xue Za Zhi. (2014) 31:196–200. 10.3760/cma.j.issn.1003-9406.2014.02.015 24711031

[59] Zhou C Zhong J Zou B Fang L Chen J Deng X. Meta-analyses of comparative efficacy of antidepressant medications on peripheral BDNF concentration in patients with depression. PLoS ONE. (2017) 12:e0172270. 10.1371/journal.pone.0172270 28241064 PMC5328267

[60] Başterzi AD Yazici K Aslan E Delialioglu N Taşdelen B Tot Acar S. Effects of fluoxetine and venlafaxine on serum brain derived neurotrophic factor levels in depressed patients. Prog Neuropsychopharmacol Biol Psychiatry. (2009) 33:281–5. 10.1016/j.pnpbp.2008.11.016 19110026

[61] Freire TF Rocha NS Fleck MP Combining ECT with pharmacological treatment of depressed inpatients in a naturalistic study is not associated with serum BDNF level increase. J Psychiatr Res. (2016) 76:30–7. 10.1016/j.jpsychires.2016.01.014 26871734

[62] Waisman A Liblau RS Becher B. Innate and adaptive immune responses in the CNS. Lancet Neurol. (2015) 14:945–55. 10.1016/S1474-4422(15)00141-6 26293566

[63] Patterson SL. Immune dysregulation and cognitive vulnerability in the aging brain: Interactions of microglia, IL-1β, BDNF and synaptic plasticity. Neuropharmacology. (2015) 96:11–8. 10.1016/j.neuropharm.2014.12.020 25549562 PMC4475415

[64] Jensen CJ Massie A De Keyser J. Immune players in the CNS: the astrocyte. J Neuroimmune Pharmacol. (2013) 8:824–39. 10.1007/s11481-013-9480-6 23821340

[65] Busch-Dienstfertig M Stein C. Opioid receptors and opioid peptide-producing leukocytes in inflammatory pain–basic and therapeutic aspects. Brain Behav Immun. (2010) 24:683–94. 10.1016/j.bbi.2009.10.013 19879349

[66] Edling AE Nanavati T Johnson JM Tuohy VK. Human and murine lymphocyte neurotrophin expression is confined to B cells. J Neurosci Res. (2004) 77:709–17. 10.1002/jnr.20176 15352217

[67] McSharry CP Fraser I Chaudhuri R Anderson K Bourke SJ Thomson NC. Nerve growth factor in serum and lymphocyte culture in pigeon fanciers' acute hypersensitivity pneumonitis. Chest. (2006) 130:37–42. 10.1378/chest.130.1.37 16840380

[68] Millet T Roger PL Eran N Yael BD Tehila M Talma B Role of the α7 nicotinic acetylcholine receptor and RIC-3 in the cholinergic anti-inflammatory pathway. Cent Nerv Syst Agents Med Chem. (2017) 17:90–9. 10.2174/1871524916666160829114533 27573666

[69] Raison CL Borisov AS Majer M Drake DF Pagnoni G Woolwine BJ. Activation of central nervous system inflammatory pathways by interferon-alpha: relationship to monoamines and depression. Biol Psychiatry. (2009) 65:296–303. 10.1016/j.biopsych.2008.08.010 18801471 PMC2655138

[70] Kraneveld AD de Theije CG van Heesch F Borre Y de Kivit S Olivier B. The neuro-immune axis: prospect for novel treatments for mental disorders. Basic Clin Pharmacol Toxicol. (2014) 114:128–36. 10.1111/bcpt.12154 24118847

[71] Misiak B Stanczykiewicz B Kotowicz K Rybakowski JK Samochowiec J Frydecka D. Cytokines and C-reactive protein alterations with respect to cognitive impairment in schizophrenia and bipolar disorder: A systematic review. Schizophr Res. (2018) 192:16–29. 10.1016/j.schres.2017.04.015 28416092

[72] Felger JC Lotrich FE. Inflammatory cytokines in depression: neurobiological mechanisms and therapeutic implications. Neuroscience. (2013) 246:199–229. 10.1016/j.neuroscience.2013.04.060 23644052 PMC3741070

[73] Hodes GE Pfau ML Leboeuf M Golden SA Christoffel DJ Bregman D. Individual differences in the peripheral immune system promote resilience versus susceptibility to social stress. Proc Natl Acad Sci USA. (2014) 111:16136–41. 10.1073/pnas.1415191111 25331895 PMC4234602

[74] Lotrich FE. Inflammatory cytokine-associated depression. Brain Res. (2015) 1617:113–25. 10.1016/j.brainres.2014.06.032 25003554 PMC4284141

[75] Bagnato GL Fiorenza A Cordova F Roberts WN Moore C Greco D. Clinical, autoimmune, and psychiatric parameters correlate with sleep disturbance in patients with systemic sclerosis and rheumatoid arthritis. Clin Exp Rheumatol. (2016) 34(Suppl. 100):49–55. 27192425

[76] Adinolfi LE Nevola R Rinaldi L Romano C Giordano M. Chronic hepatitis C virus infection and depression. Clin Liver Dis. (2017) 21:517–34. 10.1016/j.cld.2017.03.007 28689590

[77] Torres-Platas SG Cruceanu C Chen GG Turecki G Mechawar N. Evidence for increased microglial priming and macrophage recruitment in the dorsal anterior cingulate white matter of depressed suicides. Brain Behav Immun. (2014) 42:50–9. 10.1016/j.bbi.2014.05.007 24858659

[78] Tajfard M Latiff LA Rahimi HR Mouhebati M Esmaeily H Taghipour A. Serum inflammatory cytokines and depression in coronary artery disease. Iran Red Crescent Med J. (2014) 16:e17111. 10.5812/ircmj.17111 25237578 PMC4166097

[79] Dowlati Y Herrmann N Swardfager W Liu H Sham L Reim EK. A meta-analysis of cytokines in major depression. Biol Psychiatry. (2010) 67:446–57. 10.1016/j.biopsych.2009.09.033 20015486

[80] Passos IC Vasconcelos-Moreno MP Costa LG Kunz M Brietzke E Quevedo J. Inflammatory markers in post-traumatic stress disorder: a systematic review, meta-analysis, and meta-regression. Lancet Psychiatry. (2015) 2:1002–12. 10.1016/S2215-0366(15)00309-0 26544749

[81] Jiang M Qin P Yang X. Comorbidity between depression and asthma via immune-inflammatory pathways: a meta-analysis. J Affect Disord. (2014) 166:22–9. 10.1016/j.jad.2014.04.027 25012406

[82] Krause DL Riedel M Müller N Weidinger E Schwarz MJ Myint AM. Effects of antidepressants and cyclooxygenase-2 inhibitor on cytokines and kynurenines in stimulated in vitro blood culture from depressed patients. Inflammopharmacology. (2012) 20:169–76. 10.1007/s10787-011-0112-6 22237484

[83] Zou W Feng R Yang Y. Changes in the serum levels of inflammatory cytokines in antidepressant drug-naïve patients with major depression. PLoS ONE. (2018) 13:e0197267. 10.1371/journal.pone.0197267 29856741 PMC5983476

[84] Tang M Lin W Pan Y Guan X Li Y. Hippocampal neurogenesis dysfunction linked to depressive-like behaviors in a neuroinflammation induced model of depression. Physiol Behav. (2016) 161:166–73. 10.1016/j.physbeh.2016.04.034 27106565

[85] Schiepers OJ Wichers MC Maes M. Cytokines and major depression. Prog Neuropsychopharmacol Biol Psychiatry. (2005) 29:201–17. 10.1016/j.pnpbp.2004.11.003 15694227

[86] Gadek-Michalska A Tadeusz J Rachwalska P Bugajski J. Cytokines, prostaglandins and nitric oxide in the regulation of stress-response systems. Pharmacol Rep. (2013) 65:1655–62. 10.1016/S1734-1140(13)71527-5 24553014

[87] Borsini A Zunszain PA Thuret S Pariante CM. The role of inflammatory cytokines as key modulators of neurogenesis. Trends Neurosci. (2015) 38:145–57. 10.1016/j.tins.2014.12.006 25579391

[88] Eyre H Baune BT. Neuroplastic changes in depression: a role for the immune system. Psychoneuroendocrinology. (2012) 37:1397–416. 10.1016/j.psyneuen.2012.03.019 22525700

[89] Leonard B Maes M. Mechanistic explanations how cell-mediated immune activation, inflammation and oxidative and nitrosative stress pathways and their sequels and concomitants play a role in the pathophysiology of unipolar depression. Neurosci Biobehav Rev. (2012) 36:764–85. 10.1016/j.neubiorev.2011.12.005 22197082

[90] Miura H Ozaki N Sawada M Isobe K Ohta T Nagatsu T. A link between stress and depression shifts in the balance between the kynurenine and serotonin pathways of tryptophan metabolism and the etiology and pathophysiology of depression. Stress. (2008) 11:198–209. 10.1080/10253890701754068 18465467

[91] Conway-Campbell BL Pooley JR Hager GL Lightman SL. Molecular dynamics of ultradian glucocorticoid receptor action. Mol Cell Endocrinol. (2012) 348:383–93. 10.1016/j.mce.2011.08.014 21872640

[92] Sephton SE Dhabhar FS Keuroghlian AS Giese-Davis J McEwen BS Ionan AC. Depression, cortisol, and suppressed cell-mediated immunity in metastatic breast cancer. Brain Behav Immun. (2009) 23:1148–55. 10.1016/j.bbi.2009.07.007 19643176

[93] Ben Menachem-Zidon O Goshen I Kreisel T Ben Menahem Y Reinhartz E Ben Hur T Intrahippocampal transplantation of transgenic neural precursor cells overexpressing interleukin-1 receptor antagonist blocks chronic isolation-induced impairment in memory and neurogenesis. Neuropsychopharmacology. (2008) 33:2251–62. 10.1038/sj.npp.1301606 17987063

[94] Kowalska K Klimiec E Weglarczyk K Pera J Slowik A Siedlar M. Reduced ex vivo release of pro-inflammatory cytokines and elevated plasma interleukin-6 are inflammatory signatures of post-stroke delirium. J Neuroinflammation. (2018) 15:111. 10.1186/s12974-018-1156-y 29669581 PMC5907192

[95] Ganança L Oquendo MA Tyrka AR Cisneros-Trujillo S Mann JJ Sublette ME. The role of cytokines in the pathophysiology of suicidal behavior. Psychoneuroendocrinology. (2016) 63:296–310. 10.1016/j.psyneuen.2015.10.008 26546783 PMC4910882

[96] Miná VA Lacerda-Pinheiro SF Maia LC Pinheiro RF Jr Meireles CB de Souza SI. The influence of inflammatory cytokines in physiopathology of suicidal behavior. J Affect Disord. (2015) 172:219–30. 10.1016/j.jad.2014.09.057 25451421

[97] Sandrini M Vitale G Pini LA. Effect of rofecoxib on nociception and the serotonin system in the rat brain. Inflamm Res. (2002) 51:154–9. 10.1007/PL00000287 12005206

[98] Myint AM Steinbusch HW Goeghegan L Luchtman D Kim YK Leonard BE. Effect of the COX-2 inhibitor celecoxib on behavioural and immune changes in an olfactory bulbectomised rat model of depression. Neuroimmunomodulation. (2007) 14:65–71. 10.1159/000107420 17713352

[99] Müller N Schwarz MJ Dehning S Douhe A Cerovecki A Goldstein-Müller B. The cyclooxygenase-2 inhibitor celecoxib has therapeutic effects in major depression: results of a double-blind, randomized, placebo controlled, add-on pilot study to reboxetine. Mol Psychiatry. (2006) 11:680–4. 10.1038/sj.mp.4001805 16491133

[100] Akhondzadeh S Jafari S Raisi F Nasehi AA Ghoreishi A Salehi B. Clinical trial of adjunctive celecoxib treatment in patients with major depression: a double blind and placebo controlled trial. Depress Anxiety. (2009) 26:607–11. 10.1002/da.20589 19496103

[101] Abbasi SH Hosseini F Modabbernia A Ashrafi M Akhondzadeh S. Effect of celecoxib add-on treatment on symptoms and serum IL-6 concentrations in patients with major depressive disorder: randomized double-blind placebo-controlled study. J Affect Disord. (2012) 141:308–14. 10.1016/j.jad.2012.03.033 22516310

[102] Tyring S Gottlieb A Papp K Gordon K Leonardi C Wang A. Etanercept and clinical outcomes, fatigue, and depression in psoriasis: double-blind placebo-controlled randomised phase III trial. Lancet. (2006) 367:29–35. 10.1016/S0140-6736(05)67763-X 16399150

[103] Köhler CA Freitas TH Stubbs B Maes M Solmi M Veronese N. Peripheral alterations in cytokine and chemokine levels after antidepressant drug treatment for major depressive disorder: systematic review and meta-analysis. Mol Neurobiol. (2018) 55:4195–206. 10.1007/s12035-017-0632-1 28612257

[104] Réus GZ Dos Santos MA Abelaira HM Ribeiro KF Petronilho F Vuolo F. Imipramine reverses alterations in cytokines and BDNF levels induced by maternal deprivation in adult rats. Behav Brain Res. (2013) 242:40–6. 10.1016/j.bbr.2012.11.044 23238043

[105] Kim D Bae CH Jun YL Jeon H Koo S Kim S. Acupuncture alters pro-inflammatory cytokines in the plasma of maternally separated rat pups. Chin J Integr Med. (2017) 23:943–7. 10.1007/s11655-017-2827-8 28986807

[106] Rethorst CD Toups MS Greer TL Nakonezny PA Carmody TJ Grannemann BD. Pro-inflammatory cytokines as predictors of antidepressant effects of exercise in major depressive disorder. Mol Psychiatry. (2013) 18:1119–24. 10.1038/mp.2012.125 22925832 PMC3511631

[107] Wang X Zheng X Ma C Zhao L. Role of TRIF small interference RNA (siRNA) in chronic Experimental Allergic Encephalomyelitis (EAE). Med Sci Monit. (2015) 21:2583–7. 10.12659/MSM.894564 26324415 PMC4562682

[108] Kang HJ Bae KY Kim SW Shin IS Hong YJ Ahn Y. BDNF val66met polymorphism and depressive disorders in patients with acute coronary syndrome. J Affect Disord. (2016) 194:1–8. 10.1016/j.jad.2016.01.033 26795846

[109] Zhan Y Paolicelli RC Sforazzini F Weinhard L Bolasco G Pagani F. Deficient neuron-microglia signaling results in impaired functional brain connectivity and social behavior. Nat Neurosci. (2014) 17:400–6. 10.1038/nn.3641 24487234

[110] Steiner J Bielau H Brisch R Danos P Ullrich O Mawrin C. Immunological aspects in the neurobiology of suicide: elevated microglial density in schizophrenia and depression is associated with suicide. J Psychiatr Res. (2008) 42:151–7. 10.1016/j.jpsychires.2006.10.013 17174336

[111] Fang M Yuan Y Lu J Li HE Zhao M Ling EA. Scutellarin promotes microglia-mediated astrogliosis coupled with improved behavioral function in cerebral ischemia. Neurochem Int. (2016) 97:154–71. 10.1016/j.neuint.2016.04.007 27105682

[112] Tu Z Li Y Dai Y Li L Lv G Chen I. MiR-140/BDNF axis regulates normal human astrocyte proliferation and LPS-induced IL-6 and TNF-α secretion. Biomed Pharmacother. (2017) 91:899–905. 10.1016/j.biopha.2017.05.016 28501777

[113] Zhang K Wu S Li Z Zhou J. MicroRNA-211/BDNF axis regulates LPS-induced proliferation of normal human astrocyte through PI3K/AKT pathway. Biosci Rep. (2017) 37. 10.1042/BSR20170755 28790168 PMC5563540

[114] Hayley S Poulter MO Merali Z Anisman H. The pathogenesis of clinical depression: stressor- and cytokine-induced alterations of neuroplasticity. Neuroscience. (2005) 135:659–78. 10.1016/j.neuroscience.2005.03.051 16154288

[115] Koo JW Russo SJ Ferguson D Nestler EJ Duman RS. Nuclear factor-kappaB is a critical mediator of stress-impaired neurogenesis and depressive behavior. Proc Natl Acad Sci USA. (2010) 107:2669–74. 10.1073/pnas.0910658107 20133768 PMC2823860

[116] O'Léime CS Cryan JF Nolan YM. Nuclear deterrents: Intrinsic regulators of IL-1β-induced effects on hippocampal neurogenesis. Brain Behav Immun. (2017) 66:394–412. 10.1016/j.bbi.2017.07.153 28751020

[117] Wohleb ES Franklin T Iwata M Duman RS. Integrating neuroimmune systems in the neurobiology of depression. Nat Rev Neurosci. (2016) 17:497–511. 10.1038/nrn.2016.69 27277867

[118] Frühauf-Perez PK Temp FR Pillat MM Signor C Wendel AL Ulrich H. Spermine protects from LPS-induced memory deficit via BDNF and TrkB activation. Neurobiol Learn Mem. (2018) 149:135–43. 10.1016/j.nlm.2018.02.012 29458098

[119] Lotrich FE Albusaysi S Ferrell RE. Brain-derived neurotrophic factor serum levels and genotype: association with depression during interferon-α treatment. Neuropsychopharmacology. (2013) 38:985–95. 10.1038/npp.2012.263 23303061 PMC3629388

[120] Guan Z Fang J. Peripheral immune activation by lipopolysaccharide decreases neurotrophins in the cortex and hippocampus in rats. Brain Behav Immun. (2006) 20:64–71. 10.1016/j.bbi.2005.04.005 15922558

[121] Cortese GP Barrientos RM Maier SF Patterson SL Aging and a peripheral immune challenge interact to reduce mature brain-derived neurotrophic factor and activation of TrkB, PLC?1, and ERK in hippocampal synaptoneurosomes. J Neurosci. (2011) 31:4274–9. 10.1523/JNEUROSCI.5818-10.2011 21411668 PMC3086395

[122] Gibney SM McGuinness B Prendergast C Harkin A Connor TJ. Poly I:C-induced activation of the immune response is accompanied by depression and anxiety-like behaviours, kynurenine pathway activation and reduced BDNF expression. Brain Behav Immun. (2013) 28:170–81. 10.1016/j.bbi.2012.11.010 23201589

[123] Peng CH Chiou SH Chen SJ Chou YC Ku HH Cheng CK. Neuroprotection by Imipramine against lipopolysaccharide-induced apoptosis in hippocampus-derived neural stem cells mediated by activation of BDNF and the MAPK pathway. Eur Neuropsychopharmacol. (2008) 18:128–40. 10.1016/j.euroneuro.2007.05.002 17566715

[124] Zhu L Wei T Gao J Chang X He H Miao M. Salidroside attenuates lipopolysaccharide (LPS) induced serum cytokines and depressive-like behavior in mice. Neurosci Lett. (2015) 606:1–6. 10.1016/j.neulet.2015.08.025 26300543

[125] Ge L Liu L Liu H Liu S Xue H Wang X. Resveratrol abrogates lipopolysaccharide-induced depressive-like behavior, neuroinflammatory response, and CREB/BDNF signaling in mice. Eur J Pharmacol. (2015) 768:49–57. 10.1016/j.ejphar.2015.10.026 26485503

[126] Zhang K Liu J You X Kong P Song Y Cao L. P2X7 as a new target for chrysophanol to treat lipopolysaccharide-induced depression in mice. Neurosci Lett. (2016) 613:60–5. 10.1016/j.neulet.2015.12.043 26724370

[127] Yu X Jiang X Zhang X Chen Z Xu L Chen L. The effects of fisetin on lipopolysaccharide-induced depressive-like behavior in mice. Metab Brain Dis. (2016) 31:1011–21. 10.1007/s11011-016-9839-5 27209403

[128] Zhu L Nang C Luo F Pan H Zhang K Liu J. Esculetin attenuates lipopolysaccharide (LPS)-induced neuroinflammatory processes and depressive-like behavior in mice. Physiol Behav. (2016) 163:184–92. 10.1016/j.physbeh.2016.04.051 27133730

[129] Moretti M Budni J Freitas AE Neis VB Ribeiro CM de Oliveira Balen G. TNF-α-induced depressive-like phenotype and p38 (MAPK) activation are abolished by ascorbic acid treatment. Eur Neuropsychopharmacol. (2015) 25:902–12. 10.1016/j.euroneuro.2015.03.006 25836357

